# Increased expression of placenta growth factor in COPD

**DOI:** 10.1136/thx.2007.087155

**Published:** 2008-01-17

**Authors:** S-L Cheng, H-C Wang, C-J Yu, P-C Yang

**Affiliations:** 1 Department of Internal Medicine, Far Eastern Memorial Hospital, Taiwan, ROC; 2 Department of Internal Medicine, National Taiwan University Hospital, Taiwan, ROC

## Abstract

**Background:**

Vascular endothelial growth factor (VEGF) and its receptor may have an important role in the pathogenesis of emphysema. The effect of another angiogenic factor, placenta growth factor (PlGF), in chronic obstructive pulmonary disease (COPD) is unknown.

**Methods:**

The serum levels of VEGF and PlGF in patients with COPD (n = 184), smokers (n = 212) and non-smokers (n = 159) and the bronchoalveolar lavage (BAL) fluid levels of VEGF and PlGF in another group (20 patients with COPD, 18 controls) were measured. In vitro cell culture experiments were performed to investigate the effect of PlGF on VEGF.

**Results:**

The mean (SE) serum levels of PlGF were significantly higher in patients with COPD than in controls (27.1 (7.4) pg/ml vs 12.3 (5.1) pg/ml in smokers and 10.8 (6.3) pg/ml in non-smokers, p = 0.005). The levels of PlGF in BAL fluid were also significantly higher in patients with COPD than in controls (45.7 (12.3) pg/ml vs 23.9 (7.6) pg/ml, p = 0.005), associated with an increase in the cytokines tumour necrosis factor-α (TNF-α) and interleukin-8 (IL-8). In patients with COPD the levels of PlGF correlated inversely with forced expiratory volume in 1 s (FEV_1_) in serum (r = −0.59, p = 0.002) and in BAL fluid (r = −0.51, p = 0.001). While the serum levels of VEGF were the same in patients with COPD and controls, the BAL fluid levels were significantly lower in patients with COPD than in controls (127.5 (30.1) pg/ml vs 237.8 (36.1) pg/ml, p = 0.002). In cultured bronchial epithelial cells, proinflammatory cytokines induced an increase in the protein expression of both PlGF and VEGF. Continuous concomitant treatment with PlGF, TNF-α and IL-8 stimulation reduced VEGF expression and induced cell death. This phenomenon was suppressed by VEGF receptor inhibitor (CBO-P11).

**Conclusions:**

The serum and BAL fluid levels of PlGF are increased in patients with COPD and are inversely correlated with FEV_1_. Concomitant treatment with PlGF, TNF-α and IL-8 causes detrimental effects on airway epithelial cells. These data suggest that bronchial epithelial cells can express PlGF, which may contribute to the pathogenesis of COPD.

The pathogenesis of chronic obstructive pulmonary disease (COPD) is hypothesised to result from an imbalance of proteases and antiproteases in the lung.^1^ The theory proposes that increased numbers of neutrophils and macrophages, activated by cigarette smoke, produce proteases and oxidants that are responsible for the destruction of pulmonary tissues.^2^ However, in 1959, based on histological examinations of lung tissues from patients with pulmonary emphysema, Liebow noticed that the alveolar septa in centrilobular emphysema appear to be remarkably thin and almost avascular.^3^ He suggested that a reduction in the blood supply of the small precapillary blood vessels might induce the disappearance of alveolar septa. This “vascular hypothesis” of COPD has been supported by the results of a recent study in which the expression of both vascular endothelial growth factor (VEGF) and its receptor were shown to be decreased in lung tissue from patients with COPD.^4^ Besides, cigarette smoke disrupts components of VEGF_165_ and its receptor VEGFR2 with decreased expression of VEGF and its receptors in the lungs of rats and humans.^5^ The pathogenesis of COPD may therefore be more complex and multifactorial, resulting from an interaction between genetic, environmental, cigarette smoke and angiogenesis factors.

Little is known about another angiogenic growth factor called placenta growth factor (PlGF). PlGF is a 50 kDa glycosylated dimeric protein which shares significant sequence homology at the amino acid level with VEGF.^6^ Like VEGF, PlGF exhibits mitogenic activity on cultured endothelial cells and induces angiogenesis in vivo, and its effects on endothelial cells are similar to those of the potent classical angiogenic factors such as VEGF and fibroblast growth factor.^7^ Receptors for VEGF, the *fms*-like tyrosine kinase receptor (flt-1, VEGFR1) and the kinase insert domain-containing receptor (KDR, VEGFR2) are homodimeric, receptor tyrosine kinase. While VEGF binds with high affinity to both flt-1 and KDR, PlGF exhibits high affinity binding only to flt-1.^8^ Normally, PlGF mRNA is present most abundantly in the placenta, thyroid and lungs,^9^ but the biological function of PlGF in these tissues remains unclear. A previous study has shown significantly enlarged air spaces and enhanced pulmonary compliance—a situation mimicking human pulmonary emphysema—in PlGF transgenic mice.^10^ It was the first study to suggest an association between PlGF and pulmonary emphysema in mice, but whether it can be shown in humans is unknown.

A study was undertaken to elucidate the role of VEGF and PlGF in patients with COPD. We first measured the levels of VEGF and PlGF in serum and bronchoalveolar lavage (BAL) fluid from patients with COPD. We then studied the regulation of PlGF and VEGF in cultured bronchial epithelial cells after exposure to various pro-inflammatory cytokines, as well as the effects of 14 days exposure to heightened PlGF on bronchial epithelial cells.

## METHODS

### Subjects

#### Study population 1

Study population 1 consisted of 184 patients (152 men and 32 women) with smoking-related COPD. COPD was diagnosed on the basis of history, chest radiographic findings, physical examination and spirometric data, according to the American Thoracic Society guidelines.^11^ Inclusion criteria for COPD included: chronic airway symptoms and signs such as coughing, breathlessness, wheezing and chronic airway obstruction, which was defined as (1) ratio of forced expiratory volume in 1 s to forced vital capacity (FEV_1_/FVC) <70%; (2) FEV_1_ <80% of the predicted value via spirometric data; and (3) FEV_1_ reversibility after inhalation of 200 μg salbutamol of <12% of prebronchodilator FEV_1_.  Subjects were excluded if they had a history of asthma (reversibility of airflow obstruction) or malignant lung disease.

The control group included 212 asymptomatic smokers or ex-smokers (182 men and 30 women) with a smoking history of at least 10 pack-years without clinical or laboratory evidence of COPD and 159 never smokers (129 men and 30 women). All were subjects who visited the hospital for a health examination. All control subjects had normal pulmonary function (FEV_1_/FVC >70% and FEV_1_ >80% of the predicted value) and no other co-morbidities.

Peripheral blood was collected from patients with COPD and controls in heparinised syringes and centrifuged within 15 min of collection. The plasma was kept at −70°C until analysis by a technician who was blinded to the condition of the patients.

#### Study population 2

Analysis of VEGF and PlGF levels in BAL fluid was also performed in 20 patients with COPD and 18 controls (study population 2). Haemoptysis was the indication for bronchoscopy in most patients and controls, and BAL was performed if there was no evidence of bleeding or bronchial lesions. Patients with asthma, malignant lung disease or infectious processes in the airway were excluded.

### Bronchoalveolar lavage

Bronchoscopy was performed according to the standard protocol. After topical anaesthesia with 2% xylocaine, BAL was performed with four 50 ml aliquots of phosphate buffered saline instilled into the right middle lobe. The investigator made sure that no blood component was found after normal saline instillation and suction. The BAL fluid was aspirated into a siliconised glass bottle and stored on ice until processing. The chilled BAL fluid was strained through a single layer of coarse gauze to remove clumps of mucus and then spun at 400*g* for 5 min to recover cells. If red blood cells were found the samples were discarded. BAL fluid supernatant was collected and stored at −80°C until analysis.

### Measurement of VEGF, PlGF and cytokines in serum and BAL fluid

Serum and BAL fluid levels of VEGF and PlGF were assayed by a standardised sandwich enzyme-linked immunosorbent assay (ELISA) method (R&D Systems, Minneapolis, MN, USA) in duplicate according to the manufacturer’s protocol.

We calculated the concentrations of VEGF, PlGF and proinflammatory cytokines (interleukin-1β (IL-1β), tumour necrosis factor (TNF)-α, transforming growth factor (TGF)-β, epidermal growth factor (EGF) and IL-8) divided by the albumin concentration (pg/ml) in BAL fluid. The concentration of albumin in the BAL fluid was assayed by the bromocresol green method (Seiken, Tokyo, Japan). The amounts of VEGF, PlGF and the cytokines in the BAL fluid were measured using an ELISA assay (R&D Systems).

### Bronchial epithelial cell line

S-cell (ATCC number CRL-9609) human bronchial epithelial cells were grown in F12 nutrient mixture (GIBCO, Invitrogen Corporation) with 0.5 ng/ml recombinant epidermal growth factor, 500 ng/ml hydrocortisone, 0.005 mg/ml insulin, 0.035 mg/ml bovine pituitary extract, 500 nM ethanolamine, 500 nM phosphoethanolamine, 0.01 mg/ml transferrin, 6.5 ng/ml 3,3′,5-triiodothyronine, 500 ng/ml epinephrine and 0.1 ng/ml retinoic acid. Cells were grown to confluence in medium supplemented with 10% fetal calf serum which was then replaced with serum-free medium for experiments.

### Cell culture experiments

The effects of IL-1β, TNF-α, TGF-β, EGF and IL-8 (all purchased from R&D Systems) on the expression of VEGF and PlGF in lung epithelial cells were tested. These cytokines were selected because they are proinflammatory cytokines and play important roles in the pathogenesis of COPD.^12^ Final concentrations were 10 ng/ml for each cytokine.

The effect of PlGF on VEGF in lung epithelial cells was investigated by daily treatment with various agents including PlGF (50 pg/ml), TNF-α (200 pg/ml) and IL-8 (200 pg/ml), individually or concomitantly. The concentrations of cytokines were chosen because they were the mean levels of each cytokine in BAL fluid samples from patients with COPD.

The cells were treated for 14 days and VEGF expression in the conditioned media was evaluated. The conditioned media were subjected to the ELISA assay according to the manufacturer’s guidelines. Proteins (75 μg) extracted from treated cells were subject to electrophoresis on 10% gradient Bio-Tris gels (Novex, San Diego, California, USA) and transferred to PolyScreen polyvinylidine difluoride transfer membranes (Millipore Corp, Bedford, Massachusetts, USA). The primary antibodies (1:1000 dilution) were anti-human VEGF_165_ and anti-human actin (R&D Systems). The secondary antibody was anti-goat horseradish peroxidase-conjugated antibody (BioSource International, Camarillo, California, USA). VEGF_165_ was selected as being a pivotal form of the VEGF family and essential to VEGF function.

The percentage of dead cells was determined by trypan blue exclusion. Spontaneous uptake of trypan blue as a vital dye by cells was then assessed by light microscopy. Apoptosis analysis for S-cells was performed using the HTS caspase-3 assay (Oncogene Research Products, San Diego, California, USA) according to the manufacturer’s protocol at different time points. 

In a set of cells treated concomitantly with PlGF, TNF-α and IL-8, a VEGF receptor inhibitor, CBO-P11 (Calbiochem, San Diego, California, USA), was added in a concentration of 1 μM at which the binding of VEGF_165_ to VEGFR1 (IC_50_ = 700 nM) was blocked but the binding to VEGFR2 (IC_50_ = 1.3 μM) was preserved.^13^ The influence of CBO-P11 on VEGF expression and S-cell apoptosis was examined. The dose-response of CBO-P11 was also tested with concentrations from 10 nM to 1 μM on day 14 of concomitant treatment with PlGF and cytokines to determine the EC_50_ for CBO-P11 in preventing VEGF suppression and apoptosis.

### Data analysis

All the data are expressed as mean (SE) for normally distributed data. Statistical analysis was performed using SPSS 9.0 for Windows (SPSS Inc, Chicago, Illinois, USA) and analysed using post hoc testing (Scheffe test) for multiple comparisons and linear regression. A p value of <0.05 was considered statistically significant.

## RESULTS

Age, sex, smoking history and pulmonary function data of patients with COPD and control subjects (study population 1) are summarised in table 1. There were no significant differences in age or smoking history between patients with COPD and controls.

**Table 1:** Age, sex, smoking and pulmonary function data in patients with COPD and controls (study population 1)

Variables	COPD	Smokers	Non-smokers
	(n = 184)	(n = 212)	(n = 159)
Age (years)	71.9 (8.0)†	69.2 (8.0)	74.7 (3.7)
Sex (M/F)	152/32	182/30	129/30
Smoking (pack-years)	36.6 (11.5)‡	33.2 (12.8)	0
FEV_1_ (% pred)	47.2 (16.3)§	103.2 (13.7)	96.6 (15.4)
FEV_1_/FVC	45.8 (8.6)¶	79.2 (5.9)	77.5 (7.2)
Serum VEGF (pg/ml)	282.7 (13.3)**	318.4 (19.6)	296.8 (26.1)
Serum PlGF (pg/ml)	27.1 (7.4)*	12.3 (5.1)	10.8 (6.3)

All values are shown as mean (SE).

COPD, chronic obstructive pulmonary disease; FEV_1_, forced expiratory volume in 1 s; FVC, forced vital capacity; VEGF, vascular endothelial growth factor; PlGF, placenta growth factor.

*p = 0.005; †p = 0.33; ‡p = 0.12; §p<0.001; ¶p <0.001; **p = 0.35 (F test between the three groups).

**Figure 1:**
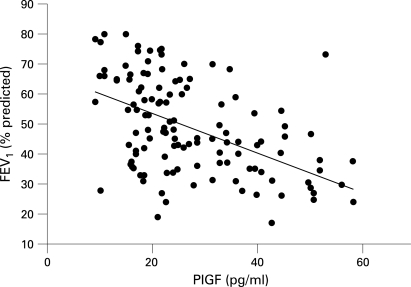
Correlation between the serum concentration of placenta growth factor (PlGF) and forced expiratory volume in 1 s (FEV_1_) percentage predicted in patients with COPD (r = −0.59, p = 0.002). The serum levels of PlGF were inversely correlated with FEV_1_.

There was no significant difference in the mean (SE) serum levels of VEGF between patients with COPD and controls (COPD: 282.7 (13.3) pg/ml; smokers: 318.4 (19.6) pg/ml; non-smoker controls: 296.8 (26.1) pg/ml, p = 0.35, F test between the three samples). However, the serum levels of PlGF were significantly higher in patients with COPD (27.1 (7.4) pg/ml than in the controls (smokers: 12.3 (5.1) pg/ml; non-smoker controls: 10.8 (6.3) pg/ml, p = 0.005). Moreover, the serum levels of PlGF correlated inversely with the value of FEV_1_ in patients with COPD (r = −0.59, p = 0.002; fig 1). Patients with higher PlGF levels tended to have lower FEV_1_ values.

**Figure 2:**
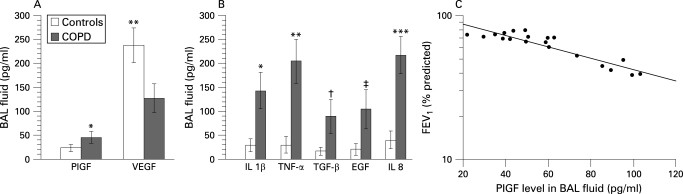
(A) Concentrations of vascular endothelial growth factor (VEGF) and placenta growth factor (PlGF) in bronchoalveolar lavage (BAL) fluid in patients with chronic obstructive pulmonary disease (COPD) and controls (*p = 0.005, **p = 0.002). Error bars are presented as standard errors. (B) Concentrations of cytokines in BAL fluid in patients with COPD and controls. IL-1β, interleukin-1β; TNF-α, tumour necrosis factor-α; TGF-β, transforming growth factor-β; EGF, epidermal growth factor; IL-8, interleukin-8. (*p = 0.003; **p = 0.001; †p = 0.005; ‡p = 0.003; ***p = 0.001). Error bars are presented as standard errors. The coefficients of variation within and between batches were 0.17 and 0.24, respectively. (C) Relationship between PlGF levels in BAL fluid and forced expiratory volume in 1 s (FEV_1_) percentage predicted in patients with COPD (r = −0.51, p = 0.001). Higher PlGF levels in BAL fluid correlated with worse lung function.

The characteristics of the 38 subjects (study population 2) receiving bronchoscopic examination and BAL are presented in table 2. The serum levels of PlGF were still significantly higher in patients with COPD (22.4 (9.7) pg/ml than in controls (13.2 (7.8) pg/ml, p<0.05), but the serum concentrations of VEGF were similar in the two groups. The concentrations of VEGF and PlGF in the BAL fluid of patients with COPD and controls are shown in fig 2A. The levels of VEGF were significantly lower in patients with COPD than in normal subjects (COPD 127.5 (30.1) pg/ml vs controls 237.8 (36.1) pg/ml, p = 0.002). However, the concentrations of PlGF in patients with COPD were higher than those of controls (COPD 45.7 (12.3) pg/ml vs controls 23.9 (7.6) pg/ml, p = 0.005; table 2). The levels of proinflammatory cytokines in BAL fluid from patients with COPD (IL-1β: 143.4 (37.9), TNF-α: 205.7 (46.1), TGF-β: 89.5 (35.1), EGF:104.3 (40.7) and IL-8: 216.8 (38.9) pg/ml) were also significantly higher than those from controls (IL-1β: 29.8 (13.6), TNF-α: 30.5 (17.2), TGF-β: 17.4 (8.5), EGF: 21.3 (11.6) and IL-8: 40.2 (18.3) pg/ml; fig 2B). Moreover, higher BAL fluid levels of PlGF were significantly correlated with worse lung function represented by FEV_1_ (r = −0.51, p = 0.001; fig 2C). The levels of VEGF in BAL fluid did not correlate with FEV_1_ (r = 0.12, p = 0.51). 

**Table 2:** Age, sex, smoking and pulmonary function data in patients with COPD and controls (study population 2)

Variables	COPD	Controls
	(n = 20)	(n = 18)
Age (years)	51.4 (12.8)†	45.6 (9.5)
Sex (M/F)	18/2	8/10
Smoking (pack-years)	18.3 (11.9)‡	15.6 (10.2)
FEV_1_ (% pred)	73.4 (7.8)§	94.8 (10.1)
FEV_1_/FVC	63.1 (6.4)¶	83.5 (4.6)
Albumin in BAL fluid (mg/ml)	0.13 (0.03)**	0.11 (0.06)
Serum VEGF (pg/ml)	298.7 (37.6)††	321.9 (30.3)
Serum PlGF (pg/ml)	22.4 (9.7)*	13.2 (7.8)

All values are shown as mean (SE).

FEV_1_, forced expiratory volume in 1 s; FVC, forced vital capacity; BAL, bronchoalveolar lavage; VEGF, vascular endothelial growth factor; PlGF, placenta growth factor.

*p<0.05; †p = 0.41; ‡p = 0.38; §p = 0.007; ¶p =  0.006; **p = 0.57; ††p = 0.47.

### Effect of cytokines on PlGF and VEGF expression

After exposure to proinflammatory cytokines (IL-1β, TNF-α, TGF-β and EGF), the expression of PlGF by S-cells increased with time. PlGF protein levels were increased 2–3-fold at 6 h and by 4–5-fold at 18 h. The levels of VEGF were also significantly increased after stimulation with the same cytokines. VEGF expression increased continuously from 6 to 18 h of culture with IL-1β, TNF-α and IL-8. However, VEGF levels cultured with TGF-β and EGF fell at 18 h.

### Effect of persistent stimulation with PlGF, TNF-α and IL-8 on VEGF expression

**Figure 3:**
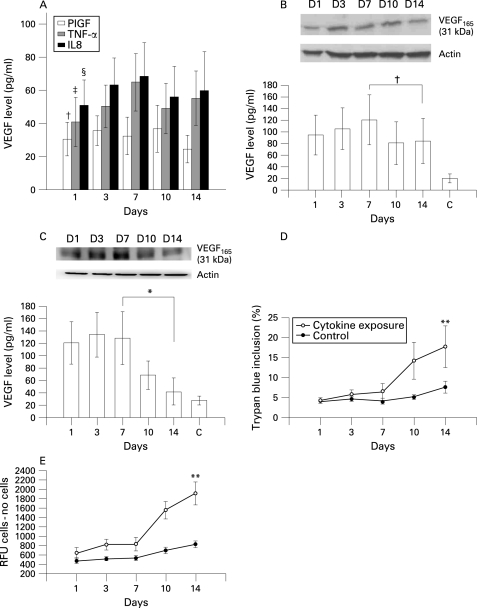
(A) Effect of persistent stimulation with exogenous PlGF, TNF-α or IL-8 on VEGF expression in S-cells. There was no significant difference in VEGF levels in individual exposures from day 1 to day 14 (†p = 0.24, ‡p = 0.31, §p = 0.16 from day 1 to day 14 individually. Error bars are presented as standard deviations. (B) Effect of persistent stimulation with a combination of TNF-α and IL-8 on VEGF expression in S-cells. Western blotting and ELISA analysis show VEGF expression from day 1 to day 14. There was no statistical difference during this period (†p = 0.43); C, control at day 14. Error bars are presented as standard deviations. (C) Effect of persistent stimulation with a combination of PlGF, TNF-α and IL-8 on VEGF suppression in S-cells. Western blotting and ELISA analysis show VEGF expression from day 1 to day 14. There was a statistically significant difference from day 7 to day 14 with gradual downregulation of VEGF expression (*p<0.05); C, control at day 14. Error bars are presented as standard deviations. (D) Percentage S-cell viability following stimulation with a combination of PlGF, TNF-α and IL-8 measured by trypan blue exclusion. The percentage of cell deaths was significantly increased after 14 days of cytokine exposure (**p<0.05 vs day 7). Error bars are presented as standard deviations. (E) Caspase-3 activity assay in S-cells after stimulation with a combination of PlGF, TNF-α and IL-8. The signal was analysed after subtracting the appropriate number of cells/buffer controls. Caspase-3 activity gradually increased after 14 days of exposure (**p<0.01 vs day 7). Error bars are presented as standard deviations. VEGF, vascular endothelial growth factor; PlGF, placenta growth factor; TNF-α, tumour necrosis factor-α; IL-8, interleukin-8; RFU, relative signal.

**Figure 4:**
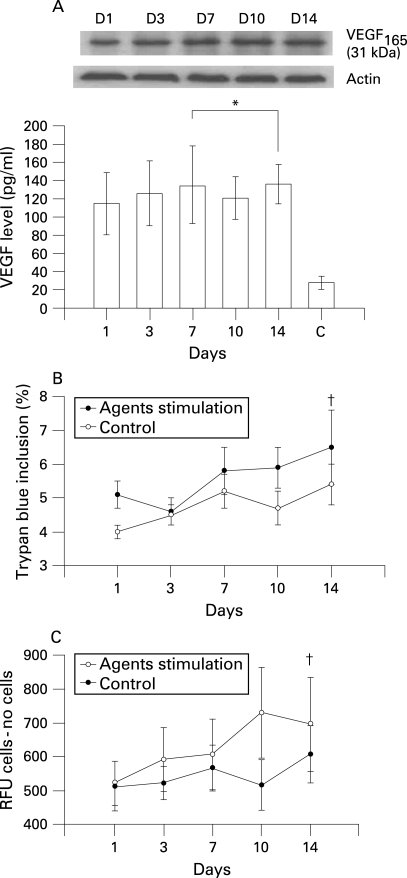
(A) Effect of persistent stimulation with a combination of PlGF, TNF-α, IL-8 and CBO-P11 1 μM/l on VEGF expression in S-cells. Western blotting and ELISA analysis show VEGF expression from day 1 to 14. There was no statistically significant difference during this period (*p = 0.52); C, control at day 14. Error bars are presented as standard deviations. (B) Percentage S-cell viability following stimulation with a combination of PlGF, TNF-α, IL-8 and CBO-P11 measured by trypan blue exclusion. The percentage of cell deaths was not significantly increased after 14 days of exposure (†p = 0.61 vs day 7). Error bars are presented as standard deviations. (C) Caspase-3 activity assay for S-cells after stimulation with a combination of PlGF, TNF-α, IL-8 and CBO-P11. The signal was analysed after subtracting the appropriate number of cells/buffer controls. Caspase-3 activity did not increase after 14 days of exposure (†p = 0.37 vs day 7). Error bars are presented as standard deviations. VEGF, vascular endothelial growth factor; PlGF, placenta growth factor; TNF-α, tumour necrosis factor-α; IL-8, interleukin-8; RFU, relative signal.

**Figure 5:**
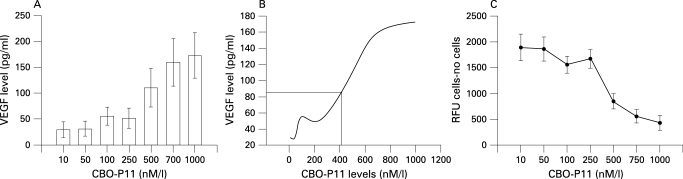
(A) S-cells were cultured with fixed concentrations of PlGF, TNF-α, IL-8 and the indicated concentrations of CBO-P11 for 14 days. The expression of VEGF in conditioned medium was measured. Error bars are presented as standard deviations of triplicate. (B) Dose-response curve of CBO-P11 showing an EC_50_ of 415 nM/l. (C) Caspase-3 activity assay of S-cells cultured with fixed concentrations of PlGF, TNF-α, IL-8 and the indicated concentrations of CBO-P11 for 14 days. Error bars are presented as standard deviations. VEGF, vascular endothelial growth factor; PlGF, placenta growth factor; TNF-α, tumour necrosis factor-α; IL-8, interleukin-8; RFU, relative signal.

The expression of VEGF after 14 days of treatment with PlGF, TNF-α or IL-8 using concentrations close to those in the BAL fluid of patients with COPD (PlGF 50 pg/ml, TNF-α 200 pg/ml and IL-8 200 pg/ml) is shown in fig 3A. With TNF-α or IL-8 stimulation alone, the expression of VEGF gradually increased during the initial 7 days and then decreased after 10–14 days of treatment. The same finding was noted after concomitant treatment with TNF-α (200 pg/ml) and IL-8 (200 pg/ml) for 10 days (fig 3B). After 10 days exposure to 50 pg/ml PlGF alone the level of VEGF decreased (fig 3A), but the expression of VEGF was significantly reduced after 10 days of concomitant treatment with PlGF (50 pg/ml), TNF-α (200 pg/ml) and IL-8 (200 pg/ml) (fig 3C). A cell viability test showed a significant increase in the percentage of dead cells after cytokine exposure for 14 days (fig 3D). Apoptosis analysis with the caspase-3 activity assay showed a considerable increase in apoptotic cells during the 14 days of stimulation (fig 3E).  The addition of VEGFR inhibitor (CBO-P11) in a concentration that mainly blocked VEGFR1 binding throughout the 14-day experimental period to the mixture of TNF-α, IL-8 and PlGF avoided the suppression of VEGF expression (fig 4A), maintained S-cell viability (fig 4B) and prevented apoptosis (fig 4C).  CBO-P11 prevented the suppression of VEGF expression in a dose-dependent manner (fig 5A) with an EC_50_ of 415 nM (fig 5B). The caspase-3 activity was also ameliorated with increasing doses of CBO-P11 (fig 5C). 

## DISCUSSION

In this study we have demonstrated the potential mechanism of PlGF in the pathogenesis of COPD. The expression of PlGF increases as a response of airway epithelial cells to proinflammatory cytokines. The sustained stimuli of cytokines and PlGF subsequently reduces VEGF expression and promotes the apoptosis of airway epithelial cells through VEGFR. The apoptosis of epithelial cells is considered essential for the pathogenesis of pulmonary emphysema.

This study has several limitations. First, the numbers of men and women were not balanced and a relatively small population was recruited, especially in study population 2 in which there were few patients with severe COPD. In Taiwan about 90% of patients with COPD and chronic smokers are men. Furthermore, it is difficult to perform bronchoscopy in patients with severe obstructive lung function because of the invasive nature of the procedure and poor baseline status. Second, BAL was performed only after definite exclusion of gross bleeding or blood clot and the samples were discarded if they were obviously haemorrhagic. However, we still could not be sure that there were no microscopic red blood cells which would affect the measurement of PlGF expression. Third, the S-cell line comprised transformed cells from primary airway epithelial cells; it is unknown whether the expression of VEGF and PlGF in S-cells could represent primary cells.

Previous studies have focused on the downregulation of VEGF and VEGFR2 in emphysematous lung tissue.^4^ In this study we have confirmed the reduced expression of VEGF in patients with COPD by measuring the levels of VEGF in BAL fluid. On the other hand, the serum and BAL fluid levels of PlGF (a VEGF homologue) increased in patients with COPD compared with smokers with normal lung function.

PlGF expression increases significantly in early gestation, peaks at around 26–30 weeks and decreases as term approaches.^14^ The biological function of PlGF after gestation and in adulthood remains unclear. The cell origins of PlGF are also unknown. Although there is evidence that synergism between VEGF and PlGF contributes to angiogenesis and plasma extravasation in pathological conditions such as in ischaemia or inflammation,^15^ we have shown that bronchial epithelial cells can express PlGF and the levels of PlGF are increased in patients with COPD.

We found that the serum concentrations of PlGF in patients with COPD were inversely correlated with FEV_1_, and higher BAL fluid levels of PlGF were seen in patients with worse airflow limitation. The increased levels of PlGF in patients with COPD may result from the stimulation of proinflammatory mediators, as we found an increase in proinflammatory cytokines in BAL fluid of patients with COPD and a bronchial epithelial cell line (S-cells) was shown to express PlGF after exposure to a variety of proinflammatory cytokines. The relationship between serum or BAL fluid levels of PlGF and lung function, even if statistically significant, does not necessarily imply a cause and effect association.

Tsao *et al*^10^ have previously shown that PlGF transgenic mice develop pathology similar to human pulmonary emphysema. Our study is the first to investigate the potential role of PlGF in the pathogenesis of COPD in humans. Based on the findings of our work, we propose the following hypothesis to explain the possible role of increased levels of PlGF in the pathogenesis of COPD. The expression of PlGF is increased secondary to the stimuli of various inflammatory mediators participating in the pathogenesis of COPD, and the increase in PlGF—acting in combination with the inflammatory mediators—leads to the death of airway epithelial and endothelial cells. Tsao *et al* showed that PlGF inhibits the proliferation of MLE-15 cells (a mouse pulmonary type II epithelial cell line) in a dose-dependent manner and significantly promotes the death of these cells.^10^ In our study, persistent treatment of PlGF, combined with TNF-α and IL-8, induced downregulation of VEGF in the human bronchial epithelial cells most likely through reducing the number of viable cells and increasing cell apoptosis. Since the concentrations of PlGF, TNF-α and IL-8 used in cell cultures were close to those measured in BAL fluid in patients with COPD, our finding could be pathologically relevant. We have shown in vitro that the chronic stimulation of epithelial cells with PlGF and other cytokines induces cell death and apoptosis, which is similar to the exposure to chronic irritants associated with lung parenchymal damage in vivo. We have also shown that this phenomenon can be abolished by a VEGFR inhibitor in a concentration mainly blocking VEGFR1. Since VEGFR1 is the receptor of PlGF, the latter is then considered to play an important role in the pathogenesis of COPD. Based on the available evidence, increased PlGF levels can be acknowledged as one factor—acting in combination with multiple cytokines—in the multifactorial pathogenesis of COPD.

We are aware that only 10–15% of smokers develop COPD. In our study, smokers with normal lung function had serum PlGF levels close to those in non-smokers despite consuming an equal amount of cigarettes to smokers with COPD. The non-COPD smokers also had fewer proinflammatory cytokines in the BAL fluid than smokers with COPD. Host factors such as genetic polymorphisms of susceptible genes may play an important role in the development of COPD. Subjects carrying genetic variants of increased function of detoxifying enzymes or a reduced response of inflammatory cytokines may generate a lower inflammatory response to cigarette smoke^16^ ^17^ so, based on our hypothesis, they have lower PlGF expression and less smoking-related damage to the lung parenchyma. It is also possible that each individual may have different capabilities for expressing PlGF under inflammatory stress, but further investigations are needed to confirm this notion.

VEGF expression in the serum did not change, unlike the expression of VEGF in the BAL fluid. Since the serum levels of VEGF or PlGF may be affected by other organs or conditions such as tissue hypoxaemia or inflammation, the expression of both factors in BAL fluid should represent more accurately the changes in lung parenchyma and reflect the real pathological mechanisms of lung diseases.

COPD is traditionally classified into chronic bronchitis and emphysema. The pathogenesis and clinical manifestations of these two subtypes may be somehow different. However, few biological markers can clearly differentiate one from the other. Previous investigators have reported increased levels of VEGF in induced sputum from patients with chronic bronchitis and decreased amounts in patients with emphysema.^18^ As far as we know, cigarette smoke reduces VEGF and VEGF receptor expression and signalling, resulting in pulmonary endothelial cell death followed by progressive disappearance of the alveolar septum.^19^ Whether downregulation of VEGF resulting from overexpression of PlGF contributes to the pathogenesis of pulmonary emphysema or whether overexpression of VEGF leading to airway and vessel remodelling contributes to chronic bronchitis still needs to be elucidated. Previous investigators have reported contradictory findings regarding VEGF expression in COPD. Kranenberg *et al*^20^ demonstrated enhanced bronchial expression of VEGF and its receptors in patients with COPD. However, Kanazawa and Yoshikawa^21^ showed that sputum levels of VEGF decreased with the severity of COPD, a finding similar to ours. These authors later reported an increase in sputum VEGF levels in patients with “bronchitis-type” COPD.^22^ The above findings reflect the nature of COPD as a disease spectrum with variable phenotypes—emphysema, chronic bronchitis, or mostly mixed; the role of angiogenesis should be different in each phenotype. Further investigations are required to recruit more patients with COPD in order to focus on specific pathological changes or phenotypes to clarify the role of these two angiogenic growth factors in COPD.

In summary, our results suggest that the levels of PlGF are increased in patients with COPD, which might contribute to the pathogenesis of COPD. Subjects with higher PlGF levels in serum and BAL fluid had worse lung function. This study also shows that human bronchial epithelial cells can express PlGF, and continuous stimulation of PlGF and proinflammatory cytokines can suppress VEGF. Persistent PlGF expression might have adverse effects on lung parenchyma by downregulating angiogenesis. The mechanisms behind the observed detrimental effects of PlGF remain to be clarified. 
